# In Vitro Antifungal Activity of New and Known Geranylated Phenols against *Phytophthora cinnamomi* Rands

**DOI:** 10.3390/ijms19061601

**Published:** 2018-05-29

**Authors:** María I. Chavez, Mauricio Soto, Franco A. Cimino, Andrés F. Olea, Luis Espinoza, Katy Díaz, Lautaro Taborga

**Affiliations:** 1 Departamento de Química, Universidad Técnica Federico Santa María, Av. España No. 1680, Valparaíso 2340000, Chile; maria.chavez@usm.cl (M.I.C.); mauricio.sotoc.13@sansano.usm.cl (M.S.); franco.cimino@alumnos.usm.cl (F.A.C.); luis.espinozac@usm.cl (L.E.); 2 Instituto de Ciencias Químicas Aplicadas, Facultad de Ingeniería, Universidad Autónoma de Chile, Santiago 8910339, Chile; andres.olea@uautonoma.cl

**Keywords:** *Phytophthora cinnamomi*, antifungal activity, geranylated phenols, Oomycetes, fungicide

## Abstract

A series of new and known geranylated phenol/methoxyphenol derivatives has been tested in vitro as inhibitor agents of mycelial growth of *Phytophthora cinnamomi*. The activity of tested compounds is correlated with the nature, number, and position of the substituent group on the aromatic ring. Results indicate that the most active geranylated derivatives are those having two hydroxyl groups (or one –OH and one –OCH_3_) attached to the aromatic ring. Interestingly, these derivatives are as active as Metalaxil^®^, a commonly used commercial fungicide. Thus, our results suggest that some of these compounds might be of agricultural interest due to their potential use as fungicides against *P. cinnamomi*. The effect of structure on fungicide activity is discussed in terms of electronic distribution on both the aromatic ring and side geranyl chain. All tested compounds have been synthesized by direct coupling of geraniol and the respective phenol. Interestingly, new digeranylated derivatives were obtained by increasing the reaction time.

## 1. Introduction

*Phytophthora* root rot, caused by the fungus Oomycete *Phytophthora cinnamomi* Rands, is a very important and destructive disease that limits the production of avocadoes and highbush blueberry plants both in Chile and worldwide [1,2]. This disease is often observed with high incidence and severity in clay soils that are saturated with water over long periods of time [3]. These conditions promote the production of sporangium and zoospores, and both may germinate and infect the plant. Water is fundamental for the dissemination of spores, while roots can be damaged by anoxia, thereby facilitating the penetration and invasion of roots by *P. cinnamomi* [4,5].

Prevention of *Phytophthora* root rot is difficult, and practices that reduce the risk of infection by this soil pathogen are a combination of strategies designed to reduce its activity or increase tolerance from the host during the critical infection period [6]. To date, host resistance is the best preventive method for reducing this disease [7]. Practices include prevention, managing water to avoid excess moisture, chemical and biological control, and resistant rootstock use [8,9].

Currently, the application of chemical fungicides and antibiotics (streptomycin and chloramphenicol) is still the most important control measure. Phosphonate fungicides and phosphorous acid are highly mobile in plants, and are supposed to control *Phytophthora* spp. by a combination of direct fungitoxic activity and the stimulation of host defense mechanisms [7,10]. However, the appearance of drug-resistant strains and increasing environmental concerns is limiting their use [11].

Thus, the quest for new chemical fungicides, preferentially derived from natural products, that are effective against *Phytophthora* spp. is a matter of current interest. In previous studies, we have reported the synthesis, structural determination, antiproliferative effect, and antifungal activity of a series of linear geranylated phenols [12,13,14]. Results indicate that there is a relationship between the chemical structure of these compounds and the inhibitory effect on mycelial growth of *Botrytis cinerea* [15,16].

Therefore, it is interesting to assess the potential application of these and other similar compounds to control *Phytophthora* root rot. Thus, in this work, the inhibitory effect of a series of geranylated phenols/methoxyphenols on mycelial growth of *Phytophthora cinnamomi* Rands has been evaluated. The results are discussed in terms of the structural features of tested compounds, trying to establish a structure–activity relationship.

## 2. Results and Discussion

**Figure 1:**
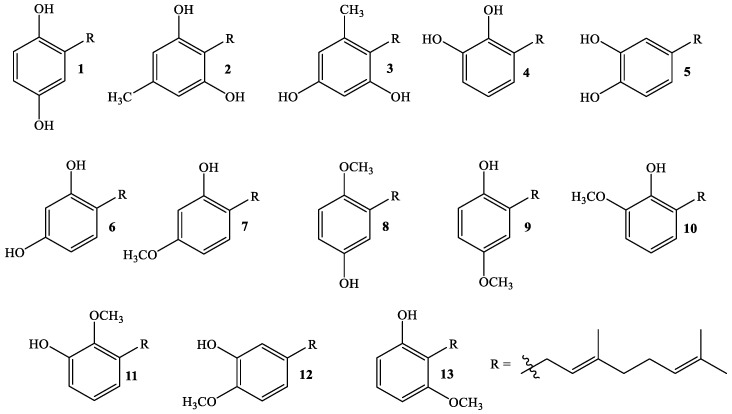
Structure of known linear geranylated phenols/methoxyphenols (Group I).

**Figure 2:**
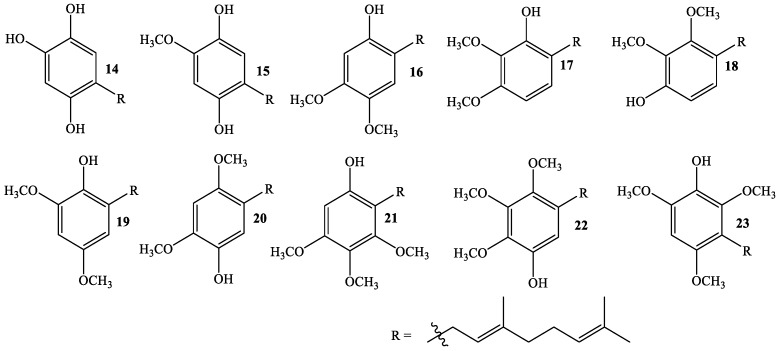
Structure of new (**14**, **17**, **18**, **21**) and other structurally related linear geranylated phenols/methoxyphenols (Group II).

**Figure 3:**
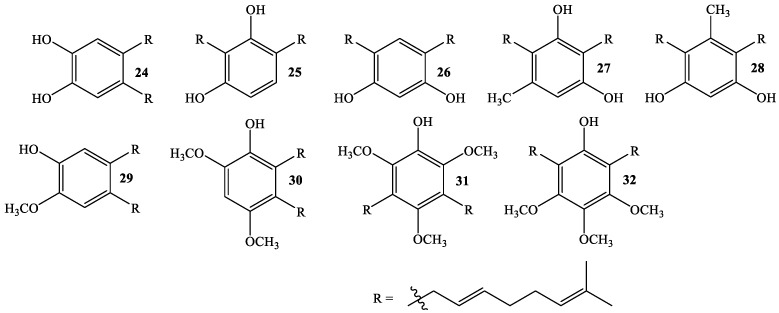
Structure of new (**24**–**26**, **29** and **32**) and other structurally related linear di-geranylated phenols/methoxyphenols (Group III).

In order to associate the antifungal activity of linear geranylated phenols/methoxyphenols against *P. cinnamomi* with their chemical structure, a large set of structurally related compounds has been used. Thus, series of known (Figure 1) and new geranylated phenols/methoxyphenols (Figure 2 and Figure 3) have been tested as inhibitors of mycelial growth of *P. cinnamomi*. All tested compounds can be classified in different groups according to nature, number, and position of substituent group on the aromatic ring (see Figure 1, Figure 2 and Figure 3). Thus, group I is formed of compounds having two oxygenated groups (two hydroxyl groups, or one of them transformed to a methoxy group) (Figure 1), whereas group II is considered to be those compounds that have three or four oxygenated groups (three hydroxyl groups, one or two of these –OH groups transformed to –OCH_3_, and one group hydroxyl group and three –OCH_3_ group) (Figure 2). Finally, group III comprises compounds with two geranyl chains attached to the aromatic ring (Figure 3).

### 2.1. Antifungal Activity of Linear Geranylated Phenols/Methoxyphenols Derivatives against P. cinnamomi In Vitro

The effect of all tested compounds (Figure 1, Figure 2 and Figure 3) on the mycelial growth of *P. cinnamomi* was determined using the radial growth rate assay, and a commercial fungicide (Metalaxil^®^) was used as the positive control. Results were expressed as a percentage of inhibition and are listed in Table 1.

Interestingly, the analysis of data in Table 1 indicates that some geranylated phenol derivatives are as active as Metalaxil^®^, a commonly used commercial fungicide. In fact, the percentages of inhibition mycelial growth obtained for compounds **1**, **3** and **6** at 150 and 250 mg/L are not significantly different from the value exhibited by the positive control, whereas at the lowest tested concentration (50 mg/L) these compounds reach a percentage of inhibition above 77%. On the other hand, at 150 mg/L compounds **2**, **4**, **5**, **7**, **8**, and **13** exhibit a percentage of 70% or higher. All these active compounds have two hydroxyl groups attached to the aromatic ring, excepting compounds **7**, **8**, and **13** in which one of the hydroxyl groups has been replaced by a methoxy group. However, the data indicate that not all isomers containing one hydroxyl and one methoxy groups are active against *P. cinnamomi*. In the case of compound **4** (a 1,2-diol), the transformation of any –OH group to –OCH_3_ brings about a dramatic decrease in activity (compounds **10** and **11**, Figure 1). The same structural change in **1** (**8** and **9**) and **6** (**7**) has a much lower impact on the activity. These results indicate that the structural distribution of substituent on the aromatic ring is the determining factor in terms of the inhibitory activity of these compounds. This effect could be attributed to induced changes in the electronic distribution of both the aromatic ring and the geranyl chain, or the production of geometrical constraints on the action site. However, the high activity shown by **13** seems to indicate that the latter possibility may be ruled out.

The introduction of a third group (–OH or –OCH_3_) in **1** brings about a slight decrease in activity (compounds **14** and **15**) and a more notable concentration effect. The attachment of an additional group to the aromatic ring (compounds **21**–**23**) decreases the activity even more. Interestingly, the activity of **10** increases from 0% to around 50% with the attachment of an additional methoxy group to the aromatic ring (compound **17**, Figure 2). This result supports the conclusion that antifungal activity depends mainly on the electronic distribution on the aromatic ring. These results indicate that a further increase of polarity on compounds of group I would induce a decrease in the antifungal activity of geranylated derivatives due to inductive effects on the aromatic ring and/or the geranyl chain.

On the other hand, digeranyl compounds exhibit much lower activity, ranging from 0% (compound **30**) to 36% (compound **27**). The main structural difference between these two compounds is that in the former both geranyl chains are in vicinal positions, whereas in the latter a hydroxyl group separates both chains. Probably, the interaction between vicinal chains decreases the activity against *P. cinnamomi*. This conclusion is in line with previous work, where it has been proposed that the biological activity of linear prenylated compounds is mainly due to the side alkyl chain [17].

Finally, it is interesting to mention that some of these compounds show antifungal activity against *Botrytis cinerea* as well [15,16,18]. A comparison of mycelial growth inhibition on both fungi indicates that most of these compounds exhibit specific activity, i.e., compounds active against *P. cinnamomi* are inactive against *B. cinerea* (**7**, **9**), and the opposite is also true (**19**, **22**, **23**, **31**). This effect may be explained in terms of different mechanisms of action depending on the type of defense of the fungus. However, it is also necessary to emphasize that there are other compounds (**1**, **2**, **3**, **16**) that are active against both pathogens, which from a commercial point of view is attractive since the same product could control the infection of more than one pathogen. The values of inhibition percentage obtained for both fungi are compared in Table S1.

In conclusion, the most efficient inhibitors of *P. cinnamomi* are geranylated derivatives, having two hydroxyl groups attached to the aromatic ring or one of them converted to a methoxy group. Increasing polarity by attaching additional –OH or –OCH_3_ groups brings about a decrease in activity. This effect is mainly attributed to changes in electronic distribution induced by substituent groups on the aromatic ring. A similar conclusion was obtained from a study of antifungal activity of a series of drimanes against a panel of human pathogenic fungi [19].

### 2.2. Chemistry

The synthesis of linear geranylated phenols is commonly carried out by direct coupling of geraniol and phenols. This reaction has received much attention because it is involved in the synthesis of phenolic terpenoids with important biological activity [12,15,16,17,20,21,22,23]. A variety of reaction conditions have been used for this coupling. For example, the syntheses of tocopherols and analogs of ubiquinones have been performed in dioxane using BF_3_·Et_2_O as a catalyst, whereas cannabigerol and related marijuana constituents were synthesized in CH_2_CI_2_ with *p*-toluenesulfonic acid as a catalyst [24]. Recently, a series of coupling reactions has been carried out in acetonitrile using a mixture of BF_3_·Et_2_O and AgNO_3_ as catalyst [15,16].

**Scheme 1:**
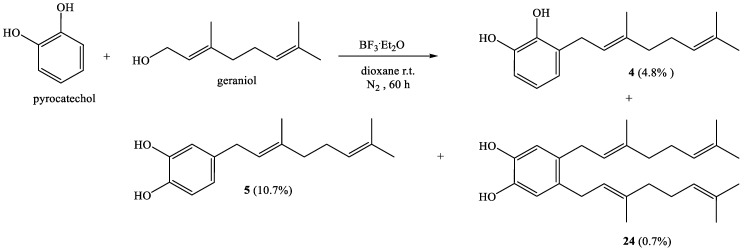
Synthesis of compounds **4**, **5**, and **24.**

**Scheme 2:**
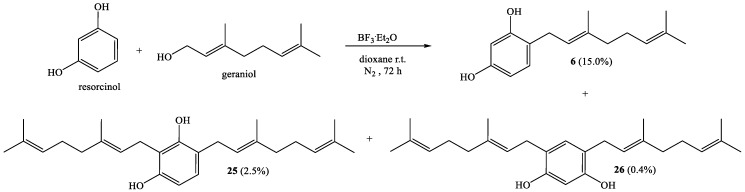
Synthesis of compounds **6, 25** and **26**.

**Scheme 3:**
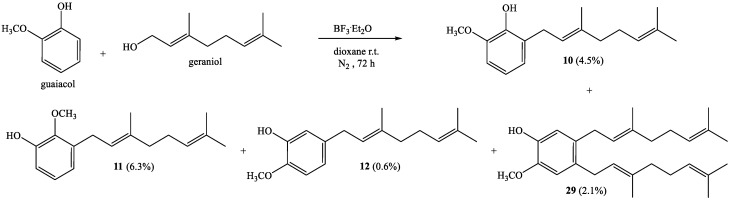
Synthesis of compounds **10**–**12** and **29**.

**Scheme 4:**
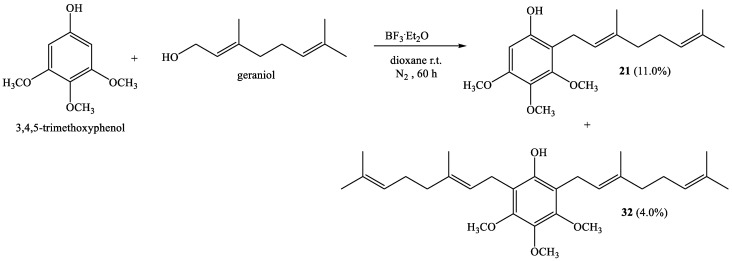
Synthesis of compounds **21** and **32**.

In previous studies, the direct coupling of geraniol to different phenols was carried out with a reaction time of 48 h [14,15,22]. In this work, the coupling reaction was performed in dioxane, using BF_3_·Et_2_O as a catalyst, and the time reaction was extended to 60–72 h. Under these conditions, new compounds **14**, **17**, **18**, **21**, **24**–**26**, **29**, and **32** have been obtained (see Scheme 1, Scheme 2, Scheme 3 and Scheme 4). The structural determination of all new compounds is detailed in the Supplementary Materials. Thus, in Section 2, *Structural Determination of New Compounds*, are shown Figures S1–S9 corresponding to the 1D NOESY and 2D HMBC spectra of compounds **14**, **17**, **18**, **21**, **24**–**26**, **29**, and **32**, respectively. In these Figures are shown the main spatial and ^1^H-^13^C HMBC correlations observed in 1D NOESY and 2D HMBC spectra for each compound.

Thus, direct coupling of pyrocatechol and geraniol for 60 h gives known compounds **4** and **5** with 4.8% and 10.7%, respectively, and new compound **24** with 0.7% yield (Scheme 1).

Geranylation between resorcinol and geraniol, with a reaction time of 72 h, gives known compound **6** [22] with 15.0% yield and new compounds **25** and **26** with 2.5% and 0.4% yields, respectively (Scheme 2).

A coupling reaction between guaiacol and geraniol for 72 h gives known compounds **10**–**12** with 4.5%, 6.3% and 0.6% yields, respectively, and new compound **29** with 2.1% (Scheme 3).

Coupling of 3,4,5-trimethoxyphenol with geraniol produces novel compounds **21** and **32** with 11.0% and 4.0% yields, respectively (Scheme 4).

The precursor, 3,4,5-trimethoxyphenol was synthesized by Baeyer–Villiger oxidation of the respective 3,4,5-trimethoxybenzaldehyde following described procedures [15,21]. This synthesis is described in detail in Section S1, Synthesis of 3,4,5-Trimethoxyphenol, of Supplementary Material.

**Scheme 5:**
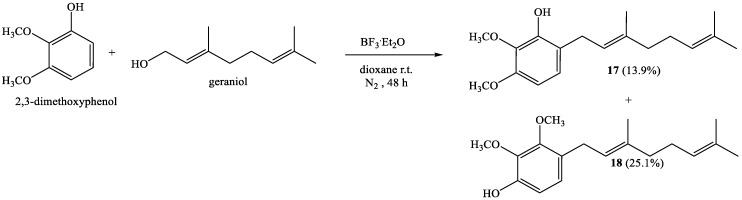
Synthesis of compounds **17** and **18**.

Direct geranylation between 2,3-dimethoxyphenol and geraniol with a reaction time of 48 h leads to novel compounds **17** and **18** with 13.9% and 25.1% yields, respectively (Scheme 5).

It is worth mentioning that the main effect of extending reaction time is formation of new digeranylated derivatives (Scheme 1, Scheme 2, Scheme 3 and Scheme 4). This change in product distribution can be attributed to the further coupling of geranylated compounds that are formed as primary products. Interestingly, the attachment of a geranyl chain induces a preferential activation of some positions on the aromatic ring. For example, coupling of **6** with geraniol leads to digeranylated compounds **25** and **26** in a 6:1 ratio (Scheme 2). Finally, the increased reactivity of these activated sites makes possible that double coupling reaction become competitive with direct geranylation of the unreacted phenol.

## 3. Materials and Methods

### 3.1. Antifungal Activity of Linear Geranylated Phenols/Methoxyphenols against Phytophthora Cinnamomi In Vitro

A radial growth rate assay on potato dextrose agar medium (PDA) was used to determine the antifungal activity of linear geranylated phenols/methoxyphenols (Figure 2 and Figure 3) [16,18]. Test compounds were added in an ethanol/water solution to Petri dishes containing a PDA medium at 50 °C. The final tested concentrations were 50, 150, and 250 mg/L for each compound. Diameter disks of 4 mm of fungus were placed at the center of the PDA agar plate. Pure solvent (1% ethanol) was used as a negative control (C−), whereas Metalaxil^®^ (ANASAC, Santiago, Chile), a commercial fungicide, was used as a positive control (C+) at the same concentrations and conditions of test compounds. Inhibition percentages of mycelial growth for each compound, after six days of incubation at 23 °C in the dark, were calculated and compared with the negative control [25].

Growth inhibition rates were calculated as percentages with the following formula, and expressed as means ± S.D.:

(1) $$\mathrm{Growth}\;\mathrm{inhibition}\;\mathrm{rate}\;\left(\%\right)=\frac{\left[\left(d_{C}-d_{O}\right)-\left(d_{S}-d_{O}\right)\right]}{\left(d_{C}-d_{O}\right)}*100$$

where *d*_0_ is the diameter of used fungus cut, *d_C_* is the diameter of blank control fungus, and *d_S_* is the diameter of compound-treated fungus. All treatments were independently performed three times in triplicate.

The results were analyzed with Statistica 7.0 software (TIBCO, Palo Alto, CA, USA), comparing the means with the value of positive control to establish significant differences (*p* < 0.05) with the Tukey HSD test.

### 3.2. General Chemistry

All reagents were purchased from commercial suppliers (Merck KGaA, Darmstadt, Germany) or Aldrich (is now Merck KGaA, Darmstadt, Germany) and used without further purification. ^1^H, ^13^C, ^13^C DEPT-135, gs 2D HSQ, C and gs 2D HMBC NMR spectra were recorded in CDCl_3_ solutions, and are referenced to the residual peaks of CHCl_3_ at *δ* = 7.26 ppm and *δ* = 77.00 ppm for ^1^H and ^13^C, respectively, on a Bruker Avance 400 Digital NMR spectrometer (Bruker, Rheinstetten, Germany), operating at 400.1 MHz for ^1^H and 100.6 MHz for ^13^C. Chemical shifts are reported in *δ* ppm and coupling constants (*J*) are given in Hz; multiplicities are reported as follows: singlet (s), doublet (d), doublet of doublets (dd), doublet of triplets (dt), triplet (t), quartet (q), and multiplet (m). IR spectra were recorded as KBr disks in a FT-IR Nicolet 6700 spectrometer (Thermo Scientific, San Jose, CA, USA) and frequencies are reported in cm^−1^. Unitary resolution mass spectra were recorded on an Agilent 5973 spectrometer (Agilent Technologies, Santa Clara, CA, USA) at 70 eV ionizing voltage coupled with a 6890 N gas chromatograph (Agilent Technologies, Santa Clara, CA, USA) equipped with a DB-5 column (30 m × 0.25 mm × 0.25 μm, and data are given as percentage of relative intensity *m*/*z* (% rel. int.). Silica gel (Merck 200–300 mesh) was used for column chromatography (CC) and silica gel plates HF_254_ for thin-layer chromatography (TLC). TLC spots were detected by heating after spraying with 25% H_2_SO_4_ in H_2_O.

### 3.3. Synthesis

In this work, compounds **4**–**6**, **10**–**12**, **14**, **21**, **24**–**26**, **29**, and **32** were synthesized by a direct coupling reaction between a phenol and geraniol, using dioxane as solvent, BF_3_·Et_2_O as a catalyst, under a nitrogen atmosphere, and with a reaction time ranging from 60 to 72 h.

#### 3.3.1. (*E*)-3-(3,7-Dimethylocta-2,6-Dienyl)Benzene-1,2-Diol (**4**); (*E*)-4-(3,7-Dimethylocta-2,6-Dienyl)Benzene-1,2-Diol (**5**); 4,5-Bis((*E*)-3,7-Dimethylocta-2,6-Dienyl)Benzene-1,2-Diol (**24**)

Coupling of pyrocatechol (1.0 g, 9.1 mmol) and geraniol (1.4 g, 9.08 mmol) was carried out in dioxane (40 mL) with BF_3_·Et_2_O (0.46 g, 3.2 mmol) as catalyst. Three fractions were obtained by CC using ethyl acetate–hexane (0:100 → 95:5): Fraction I: Compound **4** (108 mg, 4.8% yield), obtained as a reddish viscous oil; Fraction II: Compound **5** (239 mg, 10.7% yield) obtained as a reddish viscous oil; Fraction III: Compound **24** obtained as a reddish viscous oil (24.8 mg, 0.7% yield). The spectroscopic data (IR, MS and NMR) for **4** and **5** were consistent with those previously reported [14,22]. Compound **24**: ^1^H NMR (CDCl_3_, 400.1 MHz) *δ* 6.67 (s, 2H, H-3 and H-6); 5.39 (s, 2H, OH-C1 and OH-C2); 5.22 (t, *J* = 6.9 Hz, 2H, H-2′ and H-2″); 5.10 (t, *J* = 6.4 Hz, 2H, H-6′ and H-6″); 3.21 (d, *J* = 7.0 Hz, 4H, H-1′ and H-1″); 2.11-2.01 (m, 8H, H-4′, H-4″,H-5′ and H-5″); 1.68 (s, 6H, CH_3_-C3′ and CH_3_-C3″); 1.65 (s, 6H, H-8′ and H-8″); 1.60 (s, 6H, CH_3_-C7′ and CH_3_-C7″). ^13^C NMR (CDCl_3_, 100.6 MHz) *δ* 141.3 (C-2 and C-1); 135.9 (C-3′); 132.2 (C-4 and C-5); 131.4 (C-7′); 124.2 (C-6′); 123.0 (C-2′); 116.1 (C-3 and C-6); 39.7 (C-4′); 30.7 (C-1′); 26.6 (C-5′); 25.7 (C-8′); 17.7 (CH_3_-C7′); 16.1 (CH_3_-C3′). IR (cm^−1^) 3399; 2967; 2921; 2855; 1606; 1514; 1447; 1376; 1283; 1174. MS *m*/*z* (%) M^+^ 382 (4.5); 243 (17.0); 215 (21.6); 175 (100); 69 (63.6); 41 (48.9).

#### 3.3.2. (*E*)-4-(3,7-Dimethylocta-2,6-Dienyl)Benzene-1,3-Diol (**6**); 2,4-Bis((*E*)-3,7-Dimethylocta-2,6-Dienyl)Benzene-1,3-Diol (**25**); 4,6-Bis((*E*)-3,7-Dimethylocta-2,6-Dienyl)Benzene-1,3-Diol (**26**)

Coupling of resorcinol (1.0 g, 9.1 mmol) and geraniol (1.4 g, 9.1 mmol) was carried out in dioxane (20 mL) with BF_3_·Et_2_O (0.46 g, 3.2 mmol) as catalyst. Three fractions were obtained from the CC ethyl acetate–hexane (20:80): Fraction I: Compound **6** obtained as a yellow viscous oil (337.1 mg, 15.0% yield); Fraction II: Compound **25** obtained as a yellow viscous oil (86.9 mg, 10.7% yield); Fraction III: Compound **26** obtained as a yellow viscous oil (13.2 mg, 0.7% yield). The spectroscopic data (IR, MS and NMR) for **6** were consistent with those previously reported [22]. Compound **25**: ^1^H NMR (CDCl_3_, 400.1 MHz) *δ* 6.83 (d, *J* = 8.2 Hz, 1H, H-5); 6.36 (d, *J* = 8.2 Hz, 1H, H-6); 5.43 (s, 1H, HO-C1); 5.28 (m, 2H, H-2′ and H-2″); 5.06 (m, 2H, H-6′ and H-6″); 5.04 (s, 1H, HO-C3); 3.43 (d, *J* = 7.0 Hz, 2H, H-1″); 3.29 (d, *J* = 7.1 Hz, 2H, H-1′); 2.13-2.06 (m, 8H, H-4′-5′-4″-5″); 1.82 (s, 3H, CH_3_-C3″); 1.77 (s, 3H, CH_3_-C3′); 1.69 (s, 3H, H-8″); 1.68 (s, 3H, H-8′); 1.61 (s, 3H, CH_3_-C7′); 1.59 (s, 3H, CH_3_-C7″). ^13^C NMR (CDCl_3_, 100.6 MHz) *δ* 153.7 (C-3); 153.4 (C-1); 138.6 (C-3″); 138.3 (C-3′); 131.9 (C-7′ and C-7″); 127.5 (C-5); 124.0 (C-6′ and C-6″); 122.3 (C-2′); 121.7 (C-2″); 119.1 (C-4); 113.9 (C-2); 107.6 (C-6); 39.7 (C-4′ and C-4″); 29.7 (C-1′); 26.4 (C-5′ and C-5″); 25.7 (C-8′ and C-8″); 22.5 (C-1″); 17.7 (CH_3_-C7′ and CH_3_-C7″); 16.2 (CH_3_-C3″); 16.1 (CH_3_-C3′). IR (cm^−1^) 3447; 2967; 2915; 2854; 1615; 1500; 1488; 1452; 1376; 1279; 1224; 1107; 1022. MS *m*/*z* (%) M^+^ 382 (14.6); 297 (34.1); 259 (29.3); 189 (100); 135 (34.1); 69(51.2); 41 (50). Compound **26**: ^1^H NMR (CDCl_3_, 400.1 MHz) *δ* 6.78 (s, 1H, H-5); 6.33 (s, 1H, H-2); 5.29 (t, *J* = 7.1 Hz, 2H, H-2′); 5.05 (t, *J* = 5.9 Hz, 2H, H-6′); 5.04 (s, 2H, OH); 3.27 (d, *J* = 7.0 Hz, 4H, H-1′); 2.11-2.04 (m, 8H, H-5′ and H-4′); 1.78 (s, 6H, CH_3_-C3′); 1.68 (s, 6H, H-8′); 1.59 (s, 6H, CH_3_-C7′). ^13^C NMR (CDCl_3_, 100.6 MHz) *δ* 153.8 (C-1 and C-3); 138.2 (C-3′); 131.9 (C-7′); 130.9 (C-5); 123.8 (C-6′); 122.3 (C-2′); 118.7 (C-4 and C-6); 103.8 (C-2); 39.7 (C-4′); 29.3 (C-1′); 26.4 (C-5′); 25.7 (C-8′); 17.7 (CH_3_-C7′); 16.2 (CH_3_-C3′). IR (cm^−1^): 3347; 2967; 2923; 2857; 1959; 1620; 1505; 1443; 1375; 1157; 1098. MS *m*/*z* (%): M^+^ 382 (20.4); 365 (100); 259 (61.3); 207 (45.2); 69 (36.6); 41 (40.9).

#### 3.3.3. (*E*)-2-(3,7-Dimethylocta-2,6-Dienyl)-6-Methoxyphenol (**10**); (*E*)-3-(3,7-Dimethylocta-2,6-Dienyl)-2-Methoxyphenol (**11**); (*E*)-5-(3,7-Dimethylocta-2,6-Dienyl)-2-Methoxyphenol (**12**); 4,5-Bis((*E*)-3,7-Dimethylocta-2,6-Dienyl)-2-Methoxyphenol (**29**)

Coupling of guaiacol (1.5 g, 12.1 mmol) and geraniol (0.9 g, 6.0 mmol) was carried out in dioxane (20 mL) with BF_3_·Et_2_O (0.46 g, 3.2 mmol) as the catalyst. Four fractions were obtained by CC using ethyl acetate–hexane as eluent (0:100 → 90:10). Fraction I: Compound **10** obtained as a yellow viscous oil (70.6 mg, 4.5% yield); Fraction II: Compound **11** obtained as a yellow viscous oil (98.5 mg, 6.3% yield); Fraction III: Compound **12** obtained as a yellow viscous oil (9.3 mg, 0.6% yield); Fraction IV: Compound **29** was obtained as a yellow viscous oil (99.4 mg, 2.1% yield). The spectroscopic data (IR, MS and NMR) for **10**–**12** were consistent with those previously reported [15,22]. Compound **29**: ^1^H NMR (CDCl_3_, 400.1 MHz) *δ* 6.74 (s, 1H, H-6); 6.67 (s, 1H, H-3); 5.39 (s, 1H, OH); 5.24 (t, *J* = 7.2 Hz, 2H, H-2′ and H-2″); 5.09 (t, *J* = 5.8 Hz, 2H, H-6′ and H-6″); 3.84 (s, 3H, CH_3_O); 3.26 (d, *J* = 6.9 Hz, 2H, H-1″); 3.23 (d, *J* = 7.0 Hz, 2H, H-1′); 2.12-2.08 (m, 4H, H-5′ and H-5″); 2.05-2.03 (m, 4H, H-4′ and H-4″); 1.70 (s, 6H, CH_3_-C3′ and CH_3_-C3″); 1.68 (s, 6H, H-8′ and H-8″); 1.59 (s, 6H, CH_3_-C7′ and CH_3_-C7″). ^13^C NMR (CDCl_3_, 100.6 MHz) *δ* 144.5 (C-2); 143.5 (C-1); 135.8 (C-3′ and C-3″); 132.5 (C-4); 131.4 (C-7″); 131.0 (C-7′); 131.0 (C-5); 124.3 (C-6′); 124.2 (C-6″); 123.3 (C-2″); 122.9 (C-2′); 115.2 (C-6); 111.8 (C-3); 56.0 (CH_3_O); 39.7 (C-4′ and C-4″); 31.2 (C-1″); 30.8 (C-1′); 26.8 (C-5″); 26.7 (C-5′); 25.7 (C-8′ and C-8″); 17.7 (CH_3_-C7′ and CH_3_-C7″); 16.2 (CH_3_-C3′ and CH_3_-C3″). IR (cm^−1^) 3552; 3449; 2965; 2915; 2854; 1592; 1509; 1445; 1376; 1274; 1234. MS *m*/*z* (%) M^+^ 396 (7.4); 257 (27.4); 189 (100); 137 (17.9); 69 (66.3); 41 (51.6).

#### 3.3.4. (*E*)-5-(3,7-Dimethylocta-2,6-Dienyl)Benzene-1,2,4-Triol (**14**)

Coupling of benzene-1,2,4-triol (2.0 g, 15.7 mmol) and geraniol (2.7 g, 15.7 mmol) was carried out in dioxane (20 mL) with BF_3_·Et_2_O (0.31 g, 2.2 mmol) as catalyst. One fraction was obtained by CC ethyl acetate–hexane (0:100 → 90:10), a brown viscous oil corresponding to compound **14** (28.3 mg, 1.0% yield). Compound **14**: ^1^H NMR (CDCl_3_, 400.1 MHz) *δ* 6.61 (s, 1H, H-6); 6.41 (s, 1H, H-3); 5.26 (t, *J* = 7.1 Hz, 1H, H-2′); 5.06 (t, *J* = 6.0 Hz, 1H, H-6′); 3.22 (d, *J* = 7.1 Hz, 2H, H-1′); 2.09-2.05 (m, 4H, H-4′ and 5′); 1.74 (s, 3H, CH_3_-C3′); 1.68 (s, 3H, H-8′); 1.59 (s, 3H, CH_3_-C7′). ^13^C NMR (CDCl_3_, 100.6 MHz) *δ* 148.3 (C-4); 142.7 (C-2); 138.3 (C-3′); 136.7 (C-1); 131.9 (C-7′); 123.9 (C-6′); 121.9 (C-2′); 118.9 (C-5); 116.7 (C-6); 104.1 (C-3); 39.7 (C-4′); 29.1 (C-1′); 26.4 (C-5′); 25.7 (C-8′); 17.7 (CH_3_-C7′); 16.1 (CH_3_-C3′). IR (cm^−1^) 3389; 2975; 2966; 2924; 1626; 1521; 1449; 1375; 1187; 866. MS *m*/*z* (%) M^+^ 262 (39.5); 245 (100); 191 (25.3); 137 (9.9); 69 (17.3); 41 (15.8).

#### 3.3.5. (*E*)-6-(3,7-Dimethylocta-2,6-Dienyl)-2,3-Dimethoxyphenol (**17**); (*E*)-4-(3,7-Dimethylocta-2,6-Dienyl)-2,3-Dimethoxyphenol (**18**)

Coupling of 2,3-dimethoxyphenol (1.5 g, 9.7 mmol) and geraniol (1.5 g, 9.7 mmol) was carried out in dioxane (20 mL) with BF_3_·Et_2_O (1.44 g, 10 mmol) as catalyst. Two fractions were obtained by CC eluting with ethyl acetate–hexane (0.2:19.8 → 12.0:8.0): Fraction I: Compound **17** obtained as a yellow viscous oil (236.0 mg, 13.9% yield); and Fraction II: Compound **18** obtained as a yellow viscous oil (426.0 mg, 25.1% yield). Compound **17**: ^1^H NMR (CDCl_3_, 400.1 MHz) *δ* 6.79 (d, *J* = 8.5 Hz, 1H, H-5); 6.41 (d, *J* = 8.5 Hz, 1H, H-4); 5.88 (s, 1H, OH); 5.32 (t, *J* = 7.2 Hz, 1H, H-2′); 5.12 (t, *J* = 6.5 Hz, 1H, H-6′); 3.90 (s, 3H, CH_3_O-C6); 3.84 (s, 3H, CH_3_O-C5); 3.30 (d, *J* = 7.2 Hz, 2H, H-1′); 2.12-2.09 (m, 2H, H-5′); 2.07–2.03 (m, 2H, H-4′); 1.71 (s, 3H, CH_3_-C3′); 1.69 (s, 3H, H-8′); 1.61 (s, 3H, CH_3_-C7′). ^13^C NMR (CDCl_3_, 100.6 MHz) *δ* 150.5 (C-3); 147.2 (C-1); 136.2 (C-3′); 135.4 (C-2); 131.4 (C-7′); 124.4 (C-6′); 123.5 (C-5); 122.2 (C-2′); 120.7 (C-6); 103.3 (C-4); 60.9 (CH_3_O-C2); 55.8 (CH_3_O-C3); 39.7 (C-4′); 27.5 (C-1′); 26.6 (C-5′); 25.7 (C-8′); 17.7 (CH_3_-C7′); 16.0 (CH_3_-C3′). IR (cm^−1^) 3422; 2966; 2928; 2852; 1597; 1493; 1466; 1376; 1350; 1283; 1200; 1164; 1096; 1065; 1022; 962; 810. MS *m*/*z* (%) M^+^ 290 (54.7); 247 (13.2); 221 (33.9); 205 (15.0); 189 (39.6); 167 (100: M^+^ -123 (C_9_H_15_)); 153 (15.0); 129 (28.3); 91 (13.2); 69 (17.0); 41 (20.8). Compound **18**: ^1^H NMR (CDCl_3_, 400.1 MHz) *δ* 6.78 (d, *J* = 8.4 Hz, 1H, H-5); 6.65 (d, *J* = 8.4 Hz, 1H, H-6); 5.61 (s, 1H, OH); 5.25 (t, *J* = 7.2 Hz, 1H, H-2’); 5.10 (t, *J* = 6.3 Hz, 1H, H-6’); 3.92 (s, 3H, CH_3_O-C2); 3.83 (s, 3H, CH_3_O-C3); 3.27 (d, *J* = 7.2 Hz, 2H, H-1’); 2.11-2.08 (m, 2H, H-5’); 2.06–2.04 (m, 2H, H-4’); 1.71 (s, 3H, CH_3_-C3′); 1.68 (s, 3H, H-8′); 1.60 (s, 3H, CH_3_-C7′). ^13^C NMR (CDCl_3_, 100.6 MHz) *δ* 150.6 (C-2); 147.7 (C-1); 139.7 (C-3); 135.8 (C-3′); 131.4 (C-7′); 126.9 (C-4); 124.3 (C-6′); 124.2 (C-5); 123.1 (C-2′); 110.1 (C-6); 60.7 (CH_3_O-C2); 60.3 (CH_3_O-C3); 39.7 (C-4′); 27.7 (C-1′); 26.6 (C-5′); 25.7 (C-8′); 17.6 (CH_3_-C7′); 16.0 (CH_3_-C3′). IR (cm^−1^) 3522; 2960; 2926; 2849; 2840; 1617; 1505; 1464; 1430; 1376; 1279; 1216; 1096; 1038; 971; 890; 787; 696. MS *m*/*z* (%) M^+^ 290 (11.6); 221 (9.6); 205 (13.5); 189 (11.5); 167 (100: M^+^ -123 (C_9_H_15_)); 129 (17.3); 91 (7.7); 69 (9.6); 41 (13.5).

#### 3.3.6. (*E*)-2-(3,7-Dimethylocta-2,6-Dienyl)-3,4,5-Trimethoxyphenol (**21**); 2,6-Bis((*E*)-3,7-Dimethylocta-2,6-Dienyl)-3,4,5-Trimetoxyphenol (**32**)

The synthesis of 3,4,5-trimethoxyphenol is described in detail in the Supplementary Materials, Scheme S1. Coupling of 3,4,5-trimethoxyphenol (1.03 g, 5.6 mmol) and geraniol (0.87 g, 5.6 mmol) was carried out in dioxane (20 mL) with BF_3_·Et_2_O (0.30 g, 1.8 mmol) as catalyst. Two fractions were obtained by CC using ethyl acetate–hexane (0:100 → 10:90): Fraction I: Compound **21** obtained as a brown viscous oil (197.7 mg, 11.0% yield); and Fraction II: Compound **32** obtained as a brown viscous oil (98.5 mg, 4.0% yield). Compound **21**: ^1^H NMR (CDCl_3_, 400.1 MHz) *δ* 6.25 (s, 1H, H-6); 5.21 (t, *J* = 6.7 Hz, 1H, H-2’); 5.05 (t, *J* = 6.3 Hz, 1H, H-6′); 3.84 (s, 3H, CH_3_O-C3); 3.81 (s, 3H, CH_3_O-C4); 3.79 (s, 3H, CH_3_O-C5); 3.36 (d, *J* = 7.0 Hz, 2H, H-1′), 2.08–2.02 (m, 4H, H-4′ y 5′), 1.80 (s, 3H, CH_3_-C3′), 1.67 (s, 3H, H-8′), 1.59 (s, 3H, CH_3_-C7′); ^13^C NMR (CDCl_3_, 100.6 MHz) *δ* 152.1 (C-5); 151.8 (C-3); 151.2 (C-1); 138.4 (C-3′); 136.2 (C-4); 132.0 (C-7′); 123.7 (C-6′); 122.2 (C-2′); 112.3 (C-2); 96.6 (C-6); 61.2 (CH_3_O-C3); 61.0 (CH_3_O-C4); 55.9 (CH_3_O-C5); 39.7 (C-4′); 26.4 (C-5′); 25.6 (C-8′); 22.7 (C-1′); 17.7 (CH_3_-C7′); 16.1 (CH_3_-C3′). IR (cm^−1^) 3403; 2965; 2932; 2854; 1644; 1603; 1484; 1461; 1376; 1126; 1085; 998. MS *m*/*z* (%) M^+^ 320 (54.1); 197 (100); 235 (27.1); 182 (21.4); 196 (20.5). Compound **32**: ^1^H NMR (CDCl_3_, 400.1 MHz) *δ* 5.28 (s, 1H, OH); 5.21 (t, *J* = 6.8 Hz, 2H, H-2’); 5.06 (t, *J* = 6.2 Hz, 2H, H-6′); 3.85 (s, 3H, CH_3_O-C4); 3.82 (s, 6H, CH_3_O-C3 and C5); 3.36 (d, *J* = 6.9 Hz, 4H, H-1′); 2.07–2.04 (m, 8H, H-4′ and H-5′); 1.80 (s, 6H, CH_3_-C3′); 1.66 (s, 6H, H-8′); 1.58 (s, 6H, CH_3_-C7′). ^13^C NMR (CDCl_3_, 100.6 MHz) *δ* 150.0 (C-3 and C-5); 149.5 (C-1); 140.4 (C-4); 137.0 (C-3′); 131.7 (C-7′); 124.0 (C-6′); 122.6 (C-2′); 116.9 (C-2 and C-6); 61.2 (CH_3_O-C4); 60.9 (CH_3_O-C3 and CH_3_O-C5); 39.7 (C-4′); 26.5 (C-5′); 25.7 (C-8′); 23.0 (C-1′); 17.7 (CH_3_-C7′); 16.1 (CH_3_-C3′). IR (cm^−1^) 3446; 2966; 2928; 2855; 1668; 1605; 1461; 1419; 1377; 1080; 1050; 988. MS *m*/*z* (%) M^+^ 456 (52.1); 371 (18.8); 333 (32.3); 263 (80.2); 209 (100); 69 (47.9); 41 (39.6).

## 4. Conclusions

In this work a series of geranylated phenol/methoxyphenol derivatives has been synthesized and assayed against *Phytophthora cinnamomi*. The results indicate that some of these compounds are as effective as Metalaxil^®^, a commonly used commercial fungicide, in inhibiting the mycelial growth of *P. cinnamomi*. The most efficient inhibitors are geranylated derivatives having two hydroxyl groups attached to the aromatic ring, or one of them converted to a methoxy group. This effect is mainly attributed to changes in the electronic distribution induced by substituent groups on the aromatic ring. Finally, some compounds are also active against *Botrytis cinerea*, which makes them potentially useful products for agricultural applications since they could be used to control the infection of more than one pathogen.

## Figures and Tables

**Table 1:** Effect of linear geranylated phenol/methoxyphenol derivatives on the in vitro mycelial growth of *P. cinnamomi*, measured as a percentage of inhibition.

Linear Geranylated Phenols/Methoxyphenols Derivatives	Percentage of Inhibition* on In Vitro Mycelial Growth of *P. cinnamomi* (%)		
	50 mg/L	150 mg/L	250 mg/L
**1**	87 ± 0.0	100 ± 0.0 *	100 ± 0.0 *
**2**	53 ± 8.8	78 ± 3.8	96 ± 2.5 *
**3**	86 ± 1.3	95 ± 2.5 *	96 ± 2.5 *
**4**	56 ± 2.6	81 ± 10.7	85 ± 7.5
**5**	56 ± 2.5	73 ± 6.3	84 ± 1.3
**6**	82 ± 5.5	96 ± 2.5 *	98 ± 0.0 *
**7**	62 ± 3.3	73 ± 2.5	78 ± 3.8
**8**	77 ± 5.0	72 ± 3.0	75 ± 5.0
**9**	23 ± 2.5	45 ± 2.5	56 ± 2.5
**10**	0.0 ± 0.0	0.0 ± 0.0	0.0 ± 0.0
**11**	12 ± 1.3	12 ± 1.3	20 ± 2.5
**13**	45±2.5	68 ± 1.3	70 ± 1.3
**14**	15 ± 6.3	50 ± 5.8	73 ± 8.2
**15**	26 ± 3.8	44 ± 0.0	55 ± 1.3
**16**	39 ± 0.0	47 ± 1.3	55 ± 1.3
**17**	36 ± 2.5	44 ± 0.0	59 ± 3.8
**18**	22 ± 0.0	44 ± 0.0	46 ± 2.5
**19**	32 ± 1.3	30 ± 3.8	26 ± 4.5
**20**	39 ± 0.0	43 ± 1.3	51 ± 6.3
**21**	29 ± 3.3	31 ± 2.5	33 ± 0.0
**22**	22 ± 0.0	17 ± 0.0	17 ± 0.0
**23**	33 ± 0.0	25 ± 1.3	21 ± 1.3
**25**	28 ± 1.3	23 ± 1.3	00 ± 0.0
**27**	8 ± 7.0	27 ± 2.5	36 ± 3.3
**28**	0 ± 0.0	22 ± 0.0	23 ± 1.3
**29**	2 ± 2.2	9 ± 0.0	1 ± 1.3
**30**	0 ± 0.0	0 ± 0.0	0 ± 0.0
**31**	22 ± 0.0	21 ± 1.3	14 ± 3.3
**32**	2 ± 2.5	2 ± 2.5	3 ± 2.5
C+	99 ± 2.1	99 ± 1.1	100 ± 1.3
C−	00 ± 0.0	00 ± 0.0	00 ± 0.0

Notes: The percentage of inhibition of mycelial growth was based on colony diameter measurements after six days of incubation. Each point represents the mean of at least three independent experiments ± standard deviation. C+: Metalaxil^®^; C−: Negative control. * Compounds with no significant differences in the percentage of inhibition mycelial growth (%) as compared to the positive control.

## Supplementary Materials

Supplementary materials can be found at http://www.mdpi.com/1422-0067/19/6/1601/s1.
