# Supplementary material for: In Vitro Antifungal Activity of New and Known Geranylated Phenols against Phytophthora cinnamomi Rands
Source: Int J Mol Sci. 2018 May 29;19(6):1601. doi: 10.3390/ijms19061601 (PMC6032260; doi:10.3390/ijms19061601)
Supplement: Supplementary file 1 [file ijms-19-01601-s001.pdf]

Supplementary Materials

S1. Synthesis of 3,4,5-trimethoxyphenol

This compound was synthesized by Baeyer–Villiger oxidation of the respective trimethoxybenzaldehyde and subsequent saponification of the resulting trimethoxyphenyl formate (Scheme S1).

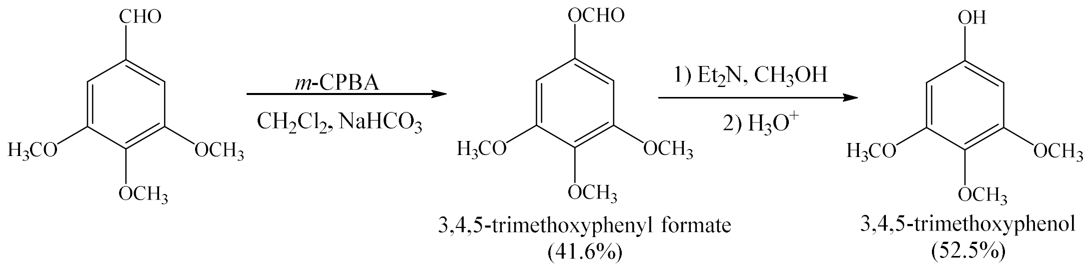

Scheme S1. Synthesis of 3,4,5-trimethoxyphenol.

In a typical reaction, *m*-chloroperoxybenzoic acid *m*-CPBA (4.45 g, 25.8 mmol) was added to a solution of 2,3,5-trimethoxybenzaldehyde (3.0 g, 15.4 mmol) and sodium bicarbonate (2.7 g, 32.1 mmol) in dichloromethane (70 mL). The mixture was stirred at room temperature until the end of the reaction was reached (~3 h). Then, the reaction mixture was filtered in a vacuum; the organic phase was washed with $NaHCO_3$ (3 × 50 mL) and water (2 × 50 mL), and dried over anhydrous Na_2SO_4. The solvent was evaporated under reduced pressure, whereas the crude was re-dissolved in CH_2Cl_2 (5 mL) and chromatographed on silica gel with hexane-ethyl acetate of increasing polarity mixtures (0:50 → 5:45). 3,4,5-trimethoxyphenyl formate was obtained as a dark brown solid (1.36 g, 41.6% yield). Three milliliters of triethylamine (2.17 g, 21 mmol) were added to a solution of 3,4,5-trimethoxyphenyl formate (1.36 g, 6.4 mmol) in methanol (75 mL) and the mixture was stirred at room temperature until the end of reaction was verified by TLC (~3 h). The solvent was evaporated under reduced pressure; the crude was diluted with ethyl acetate (20 mL) and the organic phase was washed with 5% HCl (2 × 30 mL) and water (2 × 15 mL) and dried over anhydrous Na_2SO_4. The solvent was evaporated under reduced pressure; the crude was re-dissolved in CH_2Cl_2 (5 mL) and chromatographed on silica gel with hexane–ethyl acetate of increasing polarity mixtures (0:50 → 20:30). 3,4,5-trimethoxyphenol was obtained as a dark brown oil (0.62 g, 52.5% yield). ^1H NMR ($CDCl_3$, 400.1 MHz) δ 6.09 (s, 2H, H-2 and H-6); 3.81 (s, 6H, 2 x CH_3O); 3.78 (s, 3H, CH_3O). ^{13}C NMR ($CDCl_3$, 100.6 MHz) δ 153.8 (C-3 and C-5); 152.4 (C-1); 131.9 (C-4); 93.0 (C-2 and C-6); 61.0 (CH_3O); 55.9 (2 x CH_3O). IR (cm^{-1}) 3381; 2940; 2845; 1600; 1508; 1479; 1230; 1130; 995.

S2. Structural Determination of New Compounds ***14****,* ***17****,* ***18****,* ***21****,* ***24–26****,* ***29****, and* ***32***

The chemical structure of all new compounds obtained through the synthesis described above was established by IR, MS, and mainly by NMR spectroscopy. In this section, the NMR data used to determine the chemical structure of geranylphenol derivatives (**14**, **17**, **18**, **21**, **24–26**, **29**, and **32**) are discussed in detail.

Compound **14**: In the ^1H NMR spectrum of compound **14** two singlet aromatic signals at δ_H = 6.61 and 6.41 ppm were observed, suggesting a tetrasubstituted aromatic pattern. This implies that the geranyl chain was incorporated at the C-5 position of the aromatic ring in accordance with the structure shown in Figure S1. Additionally, the signal at δ_H = 3.22 ppm (d, *J* = 7.1 Hz, 2H) assigned to H-1′ showed spatial correlations with hydrogens at δ_H = 6.61 (1H), 5.26 (t, *J* = 7.1 Hz, 1H) and 1.74 (s, 3H) ppm assigned to ArH-6, H-2′ and CH_3-C3′, respectively (Figure S.1.1). While H-1′ (d, *J* = 7.1 Hz, 2H) showed $^3J_{H\text{-}C}$ heteronuclear correlations with C-6, C-3′ and C-4 at δ_C = 116.7, 138.3 and 148.3 ppm, respectively, and $^2J_{H\text{-}C}$ correlations with C-5 and C-2′ at δ_C = 118.9 and 121.9 ppm, respectively (Figure S.1.1).

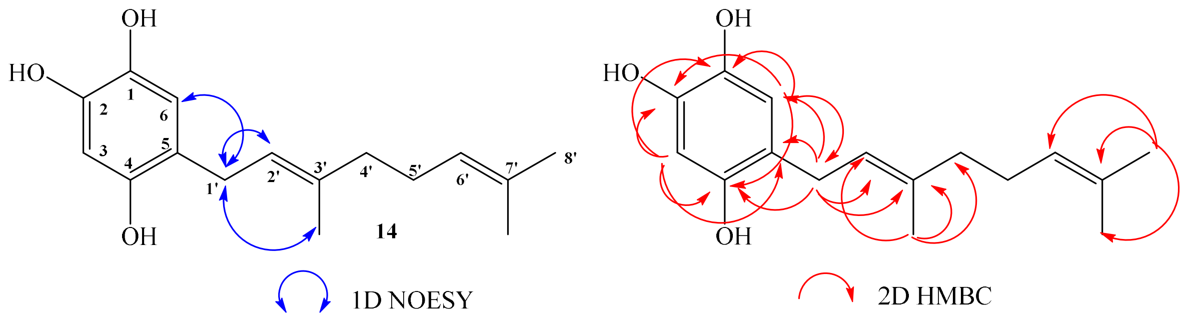

Figure S1. Mayor spatial NOE correlations (blue arrows) and main ^{1}H-^{13}C HMBC correlations (red arrows) observed for compound **14**.

Compound **17**: In the ^{1}H NMR spectrum of compound **17** two doublet aromatic signals at δ_H = 6.79 (d, J = 8.5 Hz, 1H) and 6.67 (d, J = 8.5 Hz, 1H) ppm were observed, suggesting a tetrasubstituted aromatic pattern. Additionally, two signals of methoxyl groups at δ_H = 3.90 (s, 3H) and 3.84 (s, 3H) ppm, and a signal at δ_H = 5.88 ppm (s, 1H), assigned to the OH group, were observed. However, this information is not sufficient to locate the position of the geranyl chain in the aromatic ring. However, from the 1D NOESY spectrum the signal of the OH group (s, δ_H = 5.88 ppm) showed a spatial correlation with the signal at δ_H = 3.30 ppm (d, J = 7.2 Hz, 2H), which was assigned to the H-1′ and showed a spatial correlation with the signals at δ_H = 6.79 ppm (d, J = 8.5 Hz, 1H) and δ_H = 1.71 ppm (s, 3H) assigned to ArH-5 and CH_3-C3′, respectively (Figure S.1.2) This information was consistent with the position of the geranyl chain being on the C-6 carbon of the aromatic ring. In agreement with the information observed from the 2D HMBC spectrum, the signal of H-1′ showed $^{3}J_{H\text{-}C}$ heteronuclear correlations with C-1, C-5, and C-3′ at δ_C = 147.2, 123.5, and 136.2 ppm, respectively, and $^{2}J_{H\text{-}C}$ correlations with C-6 and C-2′ at δ_C = 120.7 and 122.2 ppm, respectively (Figure S.1.2).

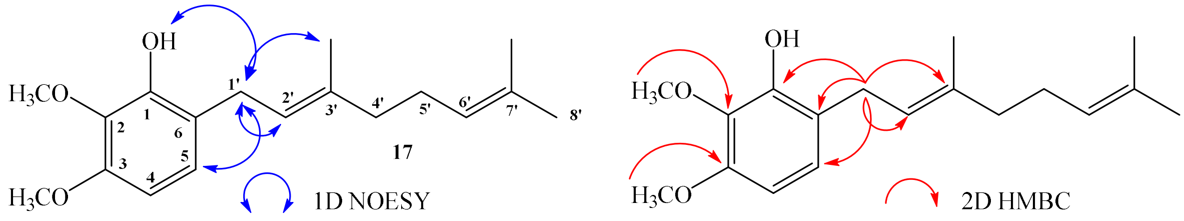

Figure S2. Mayor spatial NOE correlations (blue arrows) and main ^{1}H-^{13}C HMBC correlations (red arrows) observed for compound **17**.

Compound **18**: for the structural determination of regioisomer **18** an analysis similar to that made for **17** was carried out. In the ^{1}H NMR spectrum of compound **18** two doublet aromatic signals at δ_H = 6.78 (d, J = 8.4 Hz, 1H) and 6.65 (d, J = 8.4 Hz, 1H) ppm were observed, suggesting a tetrasubstituted aromatic pattern and an *ortho*-coupling system between these two hydrogens. Additionally, two signals of methoxyl groups at δ_H = 3.92 (s, 3H) and 3.83 (s, 3H) ppm, and a signal at δ_H = 5.61 ppm (s, 1H), assigned to the OH group, were observed. However, from the 1D NOESY spectrum, one of the methoxy groups (s, δ_H = 3.83 ppm) showed a spatial correlation with the signal at δ_H = 3.27 ppm (d, J = 7.2 Hz, 2H), which was assigned to the H-1′. This information suggests that the methoxy group is attached to the C-3 of the aromatic ring and that the geranyl chain is in an *ortho* position with respect to the methoxy group. Additionally, H-1′ showed spatial coupling with an aromatic hydrogen at δ_H = 6.78 ppm (d, J = 8.4 Hz, 1H), assigned to H-5. H-1′ also showed spatial coupling with the signal at δ_H = 1.71 ppm (s, 3H), assigned to CH_3-C3′ of the geranyl chain (Figure S.1.3). The data obtained from the 2D HMBC spectrum confirmed the proposed structure for regioisomer **18**. In this way, H-1′ showed $^{3}J_{H\text{-}C}$ heteronuclear correlations with C-3 and C-5 of aromatic ring at δ_C = 139.7 and 124.2 ppm, respectively, and with C-3′ at δ_C = 135.8 ppm of geranyl chain, while H-1′ showed $^{2}J_{H\text{-}C}$ correlation with C-4 at δ_C = 126.9 ppm and with C-2′ (geranyl chain) at δ_C = 123.1 ppm (Figure S3).

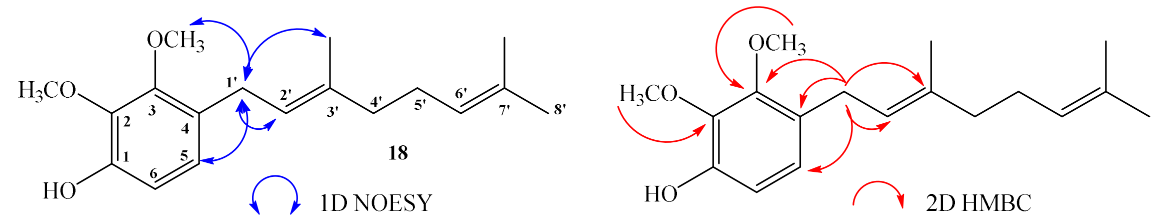

Figure S3. Mayor spatial NOE correlations (blue arrows) and main ^{1}H-^{13}C HMBC correlations (red arrows) observed for compound **18**.

Compound **21**: From the ^{1}H NMR spectrum, the presence of a single aromatic signal at $\delta_H = 6.32$ ppm (s, 1H) suggests that the aromatic system is penta-substituted and that only one geranyl chain was incorporated in a single position of the aromatic ring. Therefore, the structural determination for compound **21** does not require further analysis of spectroscopic data. However, Figure S4 shows the major spatial 1D NOESY and heteronuclear 2D HMBC correlations observed in both spectra.

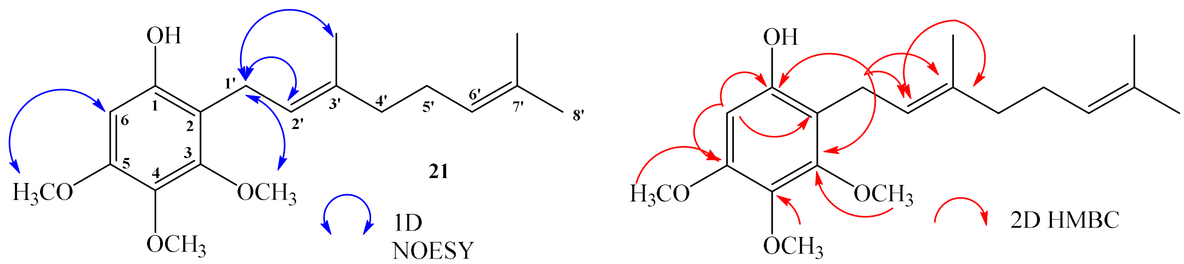

Figure S4. Mayor spatial NOE correlations (blue arrows) and main ^{1}H-^{13}C HMBC correlations (red arrows) observed for compound **21**.

Compound **24**: Spectroscopic evidence of symmetric structure and double substitution on the aromatic nucleus by geranyl chains was established from the ^{1}H-NMR spectrum by the following observations: a unique Ar–H signal appears at $\delta_H = 6.67$ ppm (s, 2H, H-3 and H-6); and the intensity of this signal was 2:4 relative to the signal at $\delta_H = 3.21$ ppm (d, $J = 7.0$ Hz, 4H, H-1′). This information suggests that geranyl chains were incorporated in carbons 4 and 5 of the aromatic ring. Finally, the complete determination of symmetrical structure was established by selective 1D NOESY experiments and 2D HMBC. From selective 1D NOESY experiments, the H-3 and H-6 signal (δ_H = 6.67 ppm) showed spatial coupling with the H-1′ and H-1′′ ($\delta_H = 3.21$ ppm) hydrogens of both geranyl chains (Figure S.1.1). The major 2D HMBC correlations, which were considered to confirm the symmetric structure of compound **24**, were a $^3J_{H\text{-}C}$ coupling observed between H-1′ with C-6 (δ_C = 116.1 ppm) and C-3′ ($\delta_C = 135.9$ ppm), while $^2J_{H\text{-}C}$ coupling was observed with C-5 and C-2′ ($\delta_C = 132.2$ and $\delta_C = 135.9$ ppm, respectively). The aromatic protons (H-3 and H-6) showed $^3J_{H\text{-}C}$ coupling with C-1′ at $\delta_C = 30.7$ ppm and $^2J_{H\text{-}C}$ with aromatic carbons C-1 and C-5 at $\delta_C = 141.3$ and 132.2 ppm, respectively. The mayor 1D NOESY and 2D HMBC correlations are shown in Figure S5.

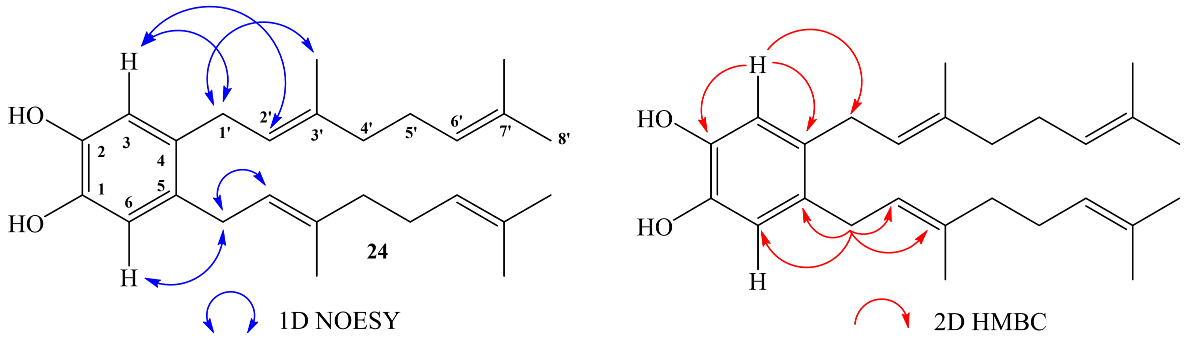

Figure S5. Mayor spatial NOE correlations (blue arrows) and main ^{1}H-^{13}C HMBC correlations (red arrows) observed for compound **24**.

Compound **25**: Spectroscopic evidence of double substitution on the aromatic nucleus by geranyl chains was established from the ^{1}H-NMR spectrum by the following observations: two Ar–H signals appeared at δ_H = 6.83 ppm (d, *J* = 8.2 Hz, 1H, H-5) and δ_H = 6.36 ppm (d, *J* = 8.2 Hz, 1H, H-4), which indicates an *ortho*-coupling between these two hydrogens and suggests in the first instance that the two geranyl chains were incorporated into the C-2 and C-4 carbons of the aromatic ring (Figure S6). However, the observation of the two singlet signals at δ_H = 5.43 and 5.04 ppm assigned to the hydrogens of the HO-C1 and HO-C3 groups, respectively, was key to determining the positions of the geranyl chains because the signal of hydrogen HO-C3 showed NOE spatial correlations with the signals at δ_H = 3.43 ppm assigned to H-1′′ (d, *J* = 7.0 Hz, 2H) and δ_H = 3.29 ppm (d, *J* = 7.1 Hz, 2H) assigned to H-1′ (Figure S6). The signal of hydrogen HO-C1 showed NOE spatial correlation with the signal at δ_H = 3.29 ppm (d, *J* = 7.1 Hz, 2H), assigned to H-1′ (Figure S6). Additionally, the H-1′′ doublet signal (δ_H = 3.43 ppm) showed spatial correlations with hydrogens at δ_H = 6.83, 5.04 and 1.77 ppm, assigned to ArH-5, HO-C3, and CH_3-C3′′, respectively (Figure S.1.6). The final confirmation of the structure for compound **25** was established from the data provided by the 2D HMBC spectrum. So the signal of H-1′′ (δ_H = 3.43 ppm) showed $^3J_{H\text{-}C}$ coupling with C-3′′ and C-3 at δ_C = 138.6 and 153.8 ppm, respectively, while H-1′′ showed $^2J_{H\text{-}C}$ with C-2 and C-2′′ at δ_C = 113.9 and 121.7 ppm, respectively, and H-1′ (δ_H = 3.29 ppm) showed $^3J_{H\text{-}C}$ coupling with C-5, C-3′, and C-1 at δ_C = 127.5, 138.3, and 153.4 ppm, respectively. H-1′ showed $^2J_{H\text{-}C}$ coupling with C-6 and C-2′ at δ_C = 119.1 and 122.3 ppm respectively (Figure S6).

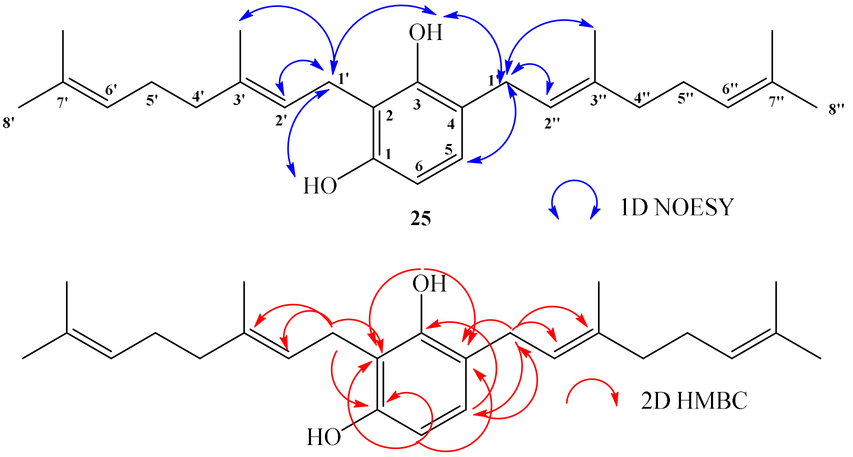

Figure S6. Major spatial NOE correlations (blue arrows) and main ^{1}H-^{13}C HMBC correlations (red arrows) observed for compound **25**.

Compound **26**: For the structural determination of compound **26** (regioisomer of **25**), a similar analysis was performed as for compound **24**. In the ^{1}H NMR spectrum of compound **26** two singlet aromatic signals at δ_H = 6.78 and 6.33 ppm were observed, suggesting a tetrasubstituted aromatic pattern. This implies that two geranyl chains were incorporated in *meta* positions each, generating a symmetrical aromatic structure (Figure S7). These signals were assigned with H-5 and H-2, whereas the signal observed at δ_H = 5.04 (s, 2H) was assigned to HO-C1 and HO-C3. Additionally, the ArH-5 signal (δ_H = 6.78 ppm, s, 1H) showed spatial correlations with hydrogens at δ_H = 3.27 (d, *J* = 7.0 Hz, 4H) and 5.29 ppm (t, *J* = 7.0 Hz, 2H) assigned to H-1′ and H-2′, respectively, while the signal at δ_H = 5.04 ppm (OH) showed spatial correlations with H-1′ and H-2′ (Figure S7). The symmetrical structure of compound **26** was established from 2D HMBC information. So, the signal of H-1′ showed $^3J_{H\text{-}C}$ coupling with C-5, C-3′ and C-1 at δ_C = 130.9, 138.2, and 153.8 ppm, respectively, and $^2J_{H\text{-}C}$ coupling with C-6 and C-2′ at δ_C = 118.7 and 122.3 ppm, respectively (Figure S7).

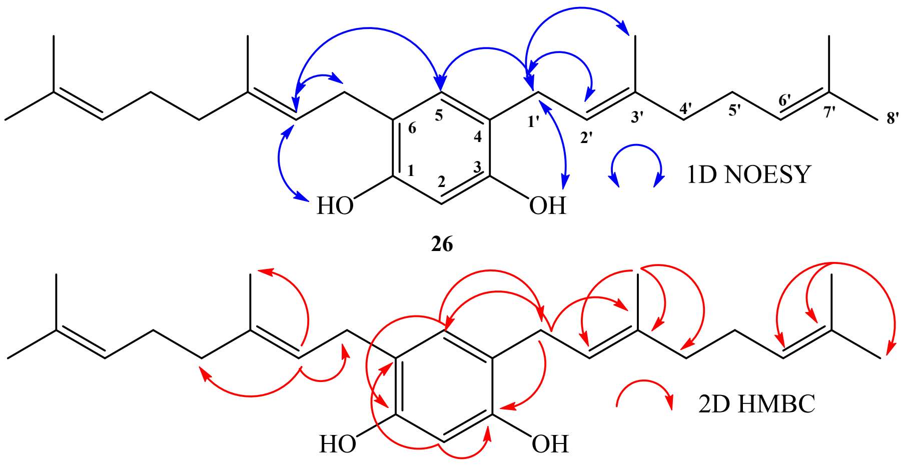

Figure S7. Mayor spatial NOE correlations (blue arrows) and main ^{1}H-^{13}C HMBC correlations (red arrows) observed for compound **26**.

Compound **29**: In the ^{1}H NMR spectrum of compound **29** two singlet aromatic signals at δ_H = 6.74 and 6.67 ppm were observed, suggesting a tetrasubstituted aromatic pattern. This implies unequivocally that two geranyl chains were incorporated in *ortho* positions between these, generating an asymmetrical aromatic structure (Figure S8). These signals were assigned to H-6 and H-3, respectively. In addition, the signals observed at δ_H = 5.39 and 3.84 ppm were assigned to the hydroxyl in C-1 (s, 1H) and the methoxyl group (s, 3H) in C-2 of the aromatic ring. From the 1D NOESY spectrum the H-6 signal (δ_H = 6.74 ppm) showed spatial coupling with a signal at δ_H = 3.26 ppm (d, *J* = 6.9 Hz, 2H), which was assigned to the H-1′′ of geranyl chain, while the H-3 signal (δ_H = 6.67 ppm) showed spatial coupling with a signal at δ_H = 3.23 ppm (d, *J* = 7.0 Hz, 2H), which was assigned to the H-1′ of another geranyl chain (Figure S8). In this way the patterns of aromatic substitution of the geranyl chains in positions C-4 and C-5 are clearly defined. In addition, the structure of compound **29** and the positions of the geranyl chains were confirmed from the 2D HMBC spectrum. Thus, the H-6 signal showed $^{3}J_{H\text{-}C}$ heteronuclear correlations with C-1′′ and C-2 at δ_C = 31.2 and 144.5 ppm, respectively, and $^{2}J_{H\text{-}C}$ correlation with C-6 at δ_C = 124.3 ppm. The H-3 signal showed $^{3}J_{H\text{-}C}$ heteronuclear correlations with C-1′ and C-1 at δ_C = 30.8 and 143.5 ppm respectively, and $^{2}J_{H\text{-}C}$ correlation with C-4 at δ_C = 132.5 ppm (Figure S8).

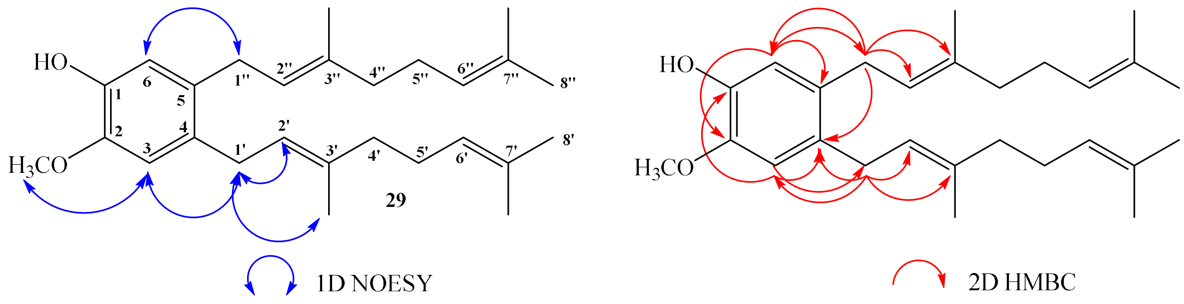

Figure S8. Mayor spatial NOE correlations (blue arrows) and main ^{1}H-^{13}C HMBC correlations (red arrows) observed for compound **29**.

Compound **32**: A similar analysis to compound **21** was carried out for compound **32**. From the ^{1}H NMR spectrum the presence of aromatic signals was not observed, suggesting that the aromatic system is fully substituted and that two geranyl chain were incorporated in the aromatic ring. Therefore, only one possible structure can be suggested for compound **32** and no further analysis of spectroscopic data is required. However, in Figure S9 the major spatial 1D NOESY and heteronuclear 2D HMBC correlations observed in both spectra are shown.

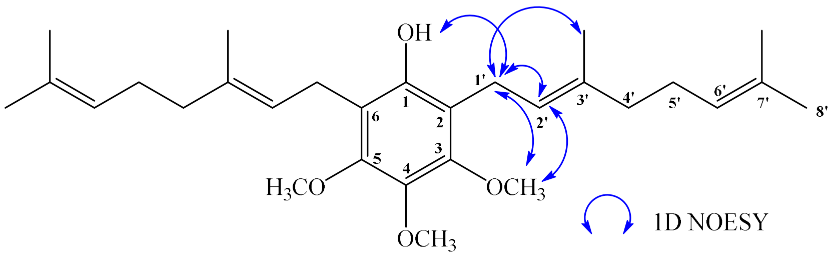

32

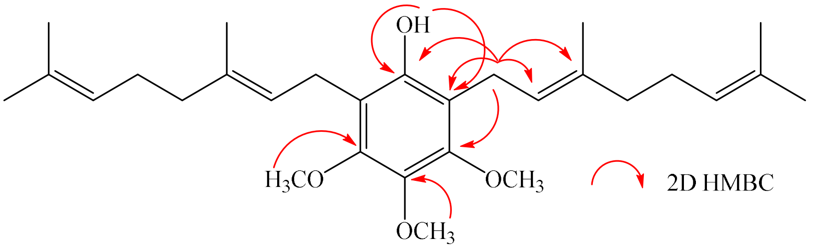

Figure S9. Mayor spatial NOE correlations (blue arrows) and main ^{1}H-^{13}C HMBC correlations (red arrows) observed for compound **32**.

Table S1. Comparison of the effect of linear geranylated phenols/methoxyphenols derivatives on in vitro mycelial growth of *P. cinnamomi* and *B. cinerea* measured as a percentage of inhibition.

**Linear geranylated phenols/methoxyphenols derivatives**	**Percentage of inhibition* on** in vitro **mycelial growth of *P. cinnamomi* and *B. cinerea.***		
	**50 mg/L**	**150 mg/L**	**250 mg/L**
**1**	87±0.0 (58 ± 2.1)	100±0.0 (82 ± 3.0)	100±0.0 (86 ± 1.3)
**2**	53±8.8 (37± 7.1)	78±3.8 (65± 8.5)	96±2.5(82± 2.3)
**3**	86±1.3 (0± 0.0)	95±2.5 (25± 7.1)	96±2.5 (63± 3.0)
**7**	62±3.3 (5±1.0)	73±2.5 (20±4.8)	78±3.8 (45±4.0)
**9**	23±2.5 (24±4.6)	45±2.5 (42±4.5)	56±2.5 (40±4.8)
**10**	0±0.0 (18±3.5)	0±0.0 (31±3.4)	0±0.0 (41±4.6)
**11**	12±1.3 (0± 0.0)	12±1.3 (4±1.4)	20±2.5 (26±5.4)
**16**	39±0.0 (49±3.3)	47±1.3 (59±3.0)	55±1.3 (79±2.1)
**19**	32±1.3 (9±2.1)	30±3.8 (46±2.8)	26±4.5 (54±4.8)
**20**	39±0.0 (32±2.2)	43±1.3 (41±4.7)	51±6.3 (71±3.8)
**22**	22±0.0 (0± 0.0)	17±0.0 (66±6.4)	17±0.0 (91±0.7)
**23**	33±0.0 (18±2.9)	25±1.3 (67±5.5)	21±1.3 (73±3.1)
**27**	8±7.0 (25± 5.5)	27±2.5 (61± 4.0)	36±3.3 (93± 2.3)
**30**	0±0.0 (0± 0.0)	0±0.0 (20±2.0)	0±0.0 (21±1.9)
**31**	22±0.0 (1±0.4)	21±1.3 (20±4.2)	14±3.3 (66±3.9)

Notes: Values in parentheses correspond to the inhibition of *B. cinerea* after 72 h. * The percentage of inhibition of mycelial growth was obtained from colony diameter measurements. Each point represents the mean of at least three independent experiments ± standard deviation.
